# Multiomics analysis to explore blood metabolite biomarkers in an Alzheimer’s Disease Neuroimaging Initiative cohort

**DOI:** 10.1038/s41598-024-56837-1

**Published:** 2024-04-02

**Authors:** Takaki Oka, Yuki Matsuzawa, Momoka Tsuneyoshi, Yoshitaka Nakamura, Ken Aoshima, Hiroshi Tsugawa, the Alzheimer’s Disease Metabolomics Consortium, Michael Weiner, Paul Aisen, Ronald Petersen, Clifford R. Jack, William Jagust, John Q. Trojanowki, Arthur W. Toga, Laurel Beckett, Robert C. Green, Andrew J. Saykin, John Morris, Leslie M. Shaw, Enchi Liu, Tom Montine, Ronald G. Thomas, Michael Donohue, Sarah Walter, Devon Gessert, Tamie Sather, Gus Jiminez, Danielle Harvey, Michael Donohue, Matthew Bernstein, Nick Fox, Paul Thompson, Norbert Schuff, Charles DeCArli, Bret Borowski, Jeff Gunter, Matt Senjem, Prashanthi Vemuri, David Jones, Kejal Kantarci, Chad Ward, Robert A. Koeppe, Norm Foster, Eric M. Reiman, Kewei Chen, Chet Mathis, Susan Landau, Nigel J. Cairns, Erin Householder, Lisa Taylor Reinwald, Virginia Lee, Magdalena Korecka, Michal Figurski, Karen Crawford, Scott Neu, Tatiana M. Foroud, Steven Potkin, Li Shen, Faber Kelley, Sungeun Kim, Kwangsik Nho, Zaven Kachaturian, Richard Frank, Peter J. Snyder, Susan Molchan, Jeffrey Kaye, Joseph Quinn, Betty Lind, Raina Carter, Sara Dolen, Lon S. Schneider, Sonia Pawluczyk, Mauricio Beccera, Liberty Teodoro, Bryan M. Spann, James Brewer, Helen Vanderswag, Adam Fleisher, Judith L. Heidebrink, Joanne L. Lord, Ronald Petersen, Sara S. Mason, Colleen S. Albers, David Knopman, Kris Johnson, Rachelle S. Doody, Javier Villanueva Meyer, Munir Chowdhury, Susan Rountree, Mimi Dang, Yaakov Stern, Lawrence S. Honig, Karen L. Bell, Beau Ances, John C. Morris, Maria Carroll, Sue Leon, Erin Householder, Mark A. Mintun, Stacy Schneider, Angela Oliver, Daniel Marson, Randall Griffith, David Clark, David Geldmacher, John Brockington, Erik Roberson, Hillel Grossman, Effie Mitsis, Leyla de Toledo-Morrell, Raj C. Shah, Ranjan Duara, Daniel Varon, Maria T. Greig, Peggy Roberts, Marilyn Albert, Chiadi Onyike, Daniel D’Agostino, Stephanie Kielb, James E. Galvin, Dana M. Pogorelec, Brittany Cerbone, Christina A. Michel, Henry Rusinek, Mony J. de Leon, Lidia Glodzik, Susan De Santi, P. Murali Doraiswamy, Jeffrey R. Petrella, Terence Z. Wong, Steven E. Arnold, Jason H. Karlawish, David Wolk, Charles D. Smith, Greg Jicha, Peter Hardy, Partha Sinha, Elizabeth Oates, Gary Conrad, Oscar L. Lopez, MaryAnn Oakley, Donna M. Simpson, Anton P. Porsteinsson, Bonnie S. Goldstein, Kim Martin, Kelly M. Makino, M. Saleem Ismail, Connie Brand, Ruth A. Mulnard, Gaby Thai, Catherine Mc Adams Ortiz, Kyle Womack, Dana Mathews, Mary Quiceno, Ramon Diaz Arrastia, Richard King, Myron Weiner, Kristen Martin Cook, Michael DeVous, Allan I. Levey, James J. Lah, Janet S. Cellar, Jeffrey M. Burns, Heather S. Anderson, Russell H. Swerdlow, Liana Apostolova, Kathleen Tingus, Ellen Woo, Daniel H. S. Silverman, Po H. Lu, George Bartzokis, Neill R. Graff Radford, Francine Parfitt, Tracy Kendall, Heather Johnson, Martin R. Farlow, Ann Marie Hake, Brandy R. Matthews, Scott Herring, Cynthia Hunt, Christopher H. van Dyck, Richard E. Carson, Martha G. MacAvoy, Howard Chertkow, Howard Bergman, Chris Hosein, Sandra Black, Bojana Stefanovic, Curtis Caldwell, Ging Yuek Robin Hsiung, Howard Feldman, Benita Mudge, Michele Assaly, Andrew Kertesz, John Rogers, Dick Trost, Charles Bernick, Donna Munic, Diana Kerwin, Marek Marsel Mesulam, Kristine Lipowski, Chuang Kuo Wu, Nancy Johnson, Carl Sadowsky, Walter Martinez, Teresa Villena, Raymond Scott Turner, Kathleen Johnson, Brigid Reynolds, Reisa A. Sperling, Keith A. Johnson, Gad Marshall, Meghan Frey, Jerome Yesavage, Joy L. Taylor, Barton Lane, Allyson Rosen, Jared Tinklenberg, Marwan N. Sabbagh, Christine M. Belden, Sandra A. Jacobson, Sherye A. Sirrel, Neil Kowall, Ronald Killiany, Andrew E. Budson, Alexander Norbash, Patricia Lynn Johnson, Thomas O. Obisesan, Saba Wolday, Joanne Allard, Alan Lerner, Paula Ogrocki, Leon Hudson, Evan Fletcher, Owen Carmichael, John Olichney, Charles DeCarli, Smita Kittur, Michael Borrie, T. Y. Lee, Rob Bartha, Sterling Johnson, Sanjay Asthana, Cynthia M. Carlsson, Steven G. Potkin, Adrian Preda, Dana Nguyen, Pierre Tariot, Adam Fleisher, Stephanie Reeder, Vernice Bates, Horacio Capote, Michelle Rainka, Douglas W. Scharre, Maria Kataki, Anahita Adeli, Earl A. Zimmerman, Dzintra Celmins, Alice D. Brown, Godfrey D. Pearlson, Karen Blank, Karen Anderson, Robert B. Santulli, Tamar J. Kitzmiller, Eben S. Schwartz, Kaycee M. Sink, Jeff D. Williamson, Pradeep Garg, Franklin Watkins, Brian R. Ott, Henry Querfurth, Geoffrey Tremont, Stephen Salloway, Paul Malloy, Stephen Correia, Howard J. Rosen, Bruce L. Miller, Jacobo Mintzer, Kenneth Spicer, David Bachman, Elizabether Finger, Stephen Pasternak, Irina Rachinsky, John Rogers, Andrew Kertesz, Dick Drost, Nunzio Pomara, Raymundo Hernando, Antero Sarrael, Susan K. Schultz, Laura L. Boles Ponto, Hyungsub Shim, Karen Elizabeth Smith, Norman Relkin, Gloria Chaing, Lisa Raudin, Amanda Smith, Kristin Fargher, Balebail Ashok Raj

**Affiliations:** 1 Department of Biotechnology and Life Science, Tokyo University of Agriculture and Technology, Tokyo, Japan; 2 Human Biology Integration Foundation, Eisai Co., Ltd., Ibaraki, Japan; 3 Microbes & Host Defense Domain, Eisai Co., Ltd., Ibaraki, Japan; 4 School of Integrative and Global Majors, University of Tsukuba, Ibaraki, Japan; 5 RIKEN Center for Sustainable Resource Science, Yokohama, Japan; 6 RIKEN Center for Integrative Medical Sciences, Yokohama, Japan; 7 Graduate School of Medical Life Science, Yokohama City University, Yokohama, Japan; 8 UC San Francisco, San Francisco, USA; 9 UC San Diego, San Diego, USA; 10 Mayo Clinic, Rochester, USA; 11 UC Berkeley, Berkeley, USA; 12 U Pennsylvania, Philadelphia, USA; 13 USC, Los Angeles, USA; 14 UC Davis, Davis, USA; 15 Brigham and Women’s Hospital, Boston, USA; 16 Harvard Medical School, Cambridge, USA; 17 Indiana University, Bloomington, USA; 18 Washington University St. Louis, St. Louis, USA; 19 University of Pennsylvania, Philadelphia, USA; 20 Janssen Alzheimer Immunotherapy, South San Francisco, USA; 21 University of Washington, Seattle, USA; 22 University of London, London, UK; 23 USC School of Medicine, Greenville, USA; 24 UCSF MRI, San Francisco, USA; 25 University of Michigan, Ann Arbor, USA; 26 University of Utah, Salt Lake City, USA; 27 Banner Alzheimer’s Institute, Phoenix, USA; 28 University of Pittsburgh, Pittsburgh, USA; 29 UPenn School of Medicine, Philadelphia, USA; 30 UC Irvine, Irvine, USA; 31 Khachaturian, Radebaugh & Associates, Inc and Alzheimer’s Association’s Ronald and Nancy Reagan’s Research Institute, Chicago, USA; 32 General Electric, Boston, USA; 33 Brown University, Providence, USA; 34 National Institute on Aging, Bethesda, USA; 35 National Institutes of Health, Bethesda, USA; 36 Oregon Health and Science University, Portland, USA; 37 University of Southern California, Los Angeles, USA; 38 University of California San Diego, La Jolla, USA; 39 Baylor College of Medicine, Houston, USA; 40 Columbia University Medical Center, New York, USA; 41 Washington University, St. Louis, USA; 42 University of Alabama Birmingham, Birmingham, USA; 43 Mount Sinai School of Medicine, New York, USA; 44 Rush University Medical Center, Chicago, USA; 45 Wien Center, Vienna, Austria; 46 Johns Hopkins University, Baltimore, USA; 47 New York University, New York, USA; 48 Duke University Medical Center, Durham, USA; 49 University of Kentucky, Lexington, USA; 50 University of Rochester Medical Center, New York, USA; 51 University of California, Irvine, USA; 52 University of Texas Southwestern Medical School, Dallas, USA; 53 Emory University, Atlanta, USA; 54 University of Kansas, Medical Center, Lawrence, USA; 55 University of California, Los Angeles, Los Angeles, USA; 56 Mayo Clinic, Jacksonville, USA; 57 Yale University School of Medicine, New Haven, USA; 58 McGill Univ., Montreal Jewish General Hospital, Montreal, Canada; 59 Sunnybrook Health Sciences, Toronto, ON Canada; 60 U.B.C. Clinic for AD & Related Disorders, Vancouver, Canada; 61 Cognitive Neurology St. Joseph’s, London, ON USA; 62 Cleveland Clinic Lou Ruvo Center for Brain Health, Las Vegas, USA; 63 Northwestern University, Evanston, USA; 64 Premiere Research Inst (Palm Beach Neurology), West Palm Beach, USA; 65 Georgetown University Medical Center, Washington, USA; 66 Stanford University, Stanford, USA; 67 Banner Sun Health Research Institute, Sun City, USA; 68 Boston University, Boston, USA; 69 Howard University, Washington, USA; 70 Case Western Reserve University, Cleveland, USA; 71 University of California, Davis Sacramento, Davis, USA; 72 Neurological Care of CNY, Syracuse, USA; 73 Parkwood Hospital, London, Canada; 74 University of Wisconsin, Madison, USA; 75 University of California, Irvine BIC, Irvine, USA; 76 Dent Neurologic Institute, Amherst, USA; 77 Ohio State University, Columbus, USA; 78 Albany Medical College, Albany, USA; 79 Hartford Hosp, Olin Neuropsychiatry Research Center, Hartford, USA; 80 Dartmouth Hitchcock Medical Center, Lebanon, USA; 81 Wake Forest University Health Sciences, Winston-Salem, USA; 82 Rhode Island Hospital, Providence, USA; 83 Butler Hospital, Providence, USA; 84 Medical University South Carolina, Charleston, USA; 85 St. Joseph’s Health Care, London, Canada; 86 Nathan Kline Institute, Orangeburg, USA; 87 University of Iowa College of Medicine, Iowa City, USA; 88 Cornell University, Ithaca, USA; 89 University of South Florida: USF Health Byrd Alzheimer’s Institute, Tampa, USA

**Keywords:** Data mining, Data processing

## Abstract

Alzheimer's disease (AD) is a neurodegenerative disease that commonly causes dementia. Identifying biomarkers for the early detection of AD is an emerging need, as brain dysfunction begins two decades before the onset of clinical symptoms. To this end, we reanalyzed untargeted metabolomic mass spectrometry data from 905 patients enrolled in the AD Neuroimaging Initiative (ADNI) cohort using MS-DIAL, with 1,304,633 spectra of 39,108 unique biomolecules. Metabolic profiles of 93 hydrophilic metabolites were determined. Additionally, we integrated targeted lipidomic data (4873 samples from 1524 patients) to explore candidate biomarkers for predicting progressive mild cognitive impairment (pMCI) in patients diagnosed with AD within two years using the baseline metabolome. Patients with lower ergothioneine levels had a 12% higher rate of AD progression with the significance of *P* = 0.012 (Wald test). Furthermore, an increase in ganglioside (GM3) and decrease in plasmalogen lipids, many of which are associated with apolipoprotein E polymorphism, were confirmed in AD patients, and the higher levels of lysophosphatidylcholine (18:1) and GM3 d18:1/20:0 showed 19% and 17% higher rates of AD progression, respectively (Wald test: *P* = 3.9 × 10^–8^ and 4.3 × 10^–7^). Palmitoleamide, oleamide, diacylglycerols, and ether lipids were also identified as significantly altered metabolites at baseline in patients with pMCI. The integrated analysis of metabolites and genomics data showed that combining information on metabolites and genotypes enhances the predictive performance of AD progression, suggesting that metabolomics is essential to complement genomic data. In conclusion, the reanalysis of multiomics data provides new insights to detect early development of AD pathology and to partially understand metabolic changes in age-related onset of AD.

## Introduction

Alzheimer's disease (AD) is the leading cause of dementia. Currently, there are > 50 million individuals with dementia worldwide, and it is estimated that this number will increase to approximately 150 million by 2060^1^. However, there is no effective pre-onset diagnostic marker or treatment for AD, and research and development of drugs or treatments for AD are mostly aimed at slowing disease progression. The pathology of AD occurs up to two decades before the onset of clinical symptoms, such as mild cognitive impairment (MCI), which is defined as the early stage of AD^2,3^. The fact that not all patients with MCI progress to AD motivates the development of biomarkers for the early detection of brain pathology, about 15% of MCI patients aged > 65 years progress to AD within 2 years^4–6^.

To date, three amyloid positron emission tomography (PET) ligands, including florbetapir, florbetaben and flutemetamol, and one tau PET ligand, fortaucipir, have been approved by the US Food and Drug Administration (FDA) as biomarkers for the diagnosis of AD^7^. Furthermore, the use of cerebrospinal fluid (CSF) biomarkers has been investigated^8^. For example, several CSF biomarkers, such as decreased amyloid-β42 (Aβ42) and increased phosphorylated tau, have been approved by the FDA and the European Medicines Agency (EMA) for the assessment of amyloid and tau pathology in AD^9^. While many studies have reported the usefulness of PET scan and CSF biomarkers^10,11^, non-invasive and less expensive diagnostic techniques are needed. Recently, there has been rapid progress in the development of blood biomarkers for the screening and clinical diagnosis of AD. Among biopsies and biomolecules, blood is one of the least invasive sources that can be obtained clinically. The plasma Aβ42/Aβ40 ratio with the APOE genotype has been validated to detect brain amyloidosis with an area under the curve of 0.93^12^; use of this biomarker is expected to become a non-invasive and inexpensive alternative to PET imaging. C_2_N Diagnostic’s PrecivityADTM^13^ test was the first blood test to receive FDA approval in 2020, and Roche’s Elecsys CSF phosphor-tau 181/Aβ42 assay also received FDA approval in 2022 to be used in clinical practice as an AD diagnostic aid.

In addition to protein-based biomarkers such as plasma Aβ42/Aβ40, the blood metabolome has the potential to become a minimally invasive and cost-effective biomarker for AD. The metabolome reflects the dysbiotic states of brain tissues and other organs. Since metabolic alterations may precede the development of clinical signs, the integration of metabolomics with existing established biomarkers may enhance early AD diagnosis and contribute to a deeper understanding of AD pathophysiological mechanisms. For example, dysfunction of glucose metabolism is a well-known phenomenon in AD and amnestic MCI, partly due to oxidative damage to the enzymes involved in glycolysis, tricarboxylic acid cycle, and adenosine triphosphate (ATP) biosynthetic pathways^14^. To reflect the oxidative damage in the brain, oxidized molecules, such as 3-nitrotyrosine, 8-hydroxy-deoxyguanosine, and 8-hydroxyguanine, which are metabolized by reactive oxygen species or reactive nitrogen species, can be used as the biomarkers^14^. Additionally, previous studies have shown that changes in lipid metabolism are strongly associated with diseased risk and progression in AD^15^. In fact, the brain is the second most lipid-rich organ after adipose tissue^16^, and lipid molecules act as scaffolds that maintain the homeostasis of molecular machinery, as well as the shape of synapses and myelin chains that are important for brain function^17^. The brain and plasma levels of docosahexaenoic acid are lower in patients with AD. Additionally, ceramide levels are elevated early in AD brains, while sphingomyelin levels are decreased, which is associated with lipid peroxidation, oxidative stress, mitochondrial dysfunction, and neuronal death^18^. The progression of neurofibrillary tangle pathology can be partly explained by the loss of sulfatide and galactosylceramide caused by reduced activity of related enzymes, such as ceramide synthase 2^19^. Ganglioside GM1 contains sialic acid and acts as a seed for Aβ binding and aggregation^20^. Moreover, many of the genetic risk factors for AD are related to lipid metabolism, including the lipid-binding apolipoprotein E (APOE), phosphatidylinositol binding clathrin assembly protein, ATP-binding cassette subfamily A members 1 and 7 (ABCA1 and ABCA7) for phospholipid and cholesterol transport, and a key regulator of cholesterol metabolism sterol regulatory-element binding protein 2^18^. Of these, APOE is highly expressed in the liver and brain, where it plays a crucial role in lipid metabolism and cholesterol transport^21^. This gene exists in three isoforms: APOE2, APOE3, and APOE4; the APOE4 variant notably increases the risk for AD^22^. In the context of AD pathophysiology, APOE is involved in the clearance of Aβ, and the APOE4 isoform is less effective in Aβ clearance, leading to an accumulation of amyloid plaques in the brain^23^. The concentration of APOE in the human central nervous system (CNS) generally increases with age. However, in cases marked by Aβ accumulation in CSF, a decrease in APOE levels has been observed, with a particularly notable reduction in the APOE4 isoform^23^. These facts indicate that metabolic alterations exist in MCI and AD patients, leading to investigations of the blood metabolome for the prediction of AD progression, especially considering the strong association of AD with metabolic dysfunctions.

In this study, we reanalyzed the raw liquid chromatography tandem mass spectrometry (LC–MS/MS) data registered in the Alzheimer's Disease Neuroimaging Initiative (ADNI), with the aim of developing biomarkers for AD, improving diagnostic methods for the early detection of AD, and optimizing clinical trial design. Mass spectrometry data were available for 905 participants (dataset name: untargeted LC–MS/MS analysis version 1.0), whereas 2,745 participants were enrolled in the ADNI. Among the studies using the ADNI data^24–26^, there was only one report describing the protocol for the analysis of human serum samples, with a brief summary of the data analysis techniques^27^. Thus, mining of hydrophilic metabolome data is valuable to the scientific community for investigating the metabolic profiles associated with AD. Since large cohort projects, such as ADNI, allow for the differentiation of background factors that are difficult to achieve with smaller datasets, the results would yield more reliable candidate biomarkers. Additionally, targeted human plasma lipidomic data from 4873 samples of 1524 individuals and single nucleotide polymorphism (SNP) data from 808 participants were included in the statistical analyses.

Using metabolomic data, we investigated metabolic signatures not only to clarify the progressive changes from healthy controls to MCI and AD but also to predict future clinical symptoms from past metabolomic data. In this study, we defined progressive MCI (pMCI) as AD diagnosed from 6 months by two years from the baseline stage, while others were classified as having sustained MCI (sMCI), where patients diagnosed as AD within 6 months were excluded. The baseline metabolome, the profile of which was obtained at the MCI stage, was examined to identify biomarkers for distinguishing pMCI from sMCI.

## Materials and methods

### Datasets

The entire approval for this study was obtained from the Eisai Ethics Committee (2017-0433). Untargeted hydrophilic metabolomics, targeted lipidomics, SNP, and the clinical data were downloaded from the ADNI database (https://adni.loni.usc.edu/) on October 12, 2021. The ADNI was launched in 2003 as a public–private partnership aiming at validating biomarkers for use in clinical treatment trials for patients with AD. The study was approved by the Institutional Review Boards at each ADNI site. Informed consent was obtained from all subjects prior to enrollment. All methods were carried out in accordance with relevant guidelines and regulations. In this study, the clinical data of APOE haplotype, age, and clinical diagnostic labels (healthy, MCI including early MCI and late MCI, and AD), were utilized. Furthermore, patients diagnosed with MCI at baseline who developed AD from 6 months by 2 years were defined as having progressive MCI (pMCI), and the others were classified as having sustained MCI (sMCI). Data from patients diagnosed as AD within 6 months from the baseline were excluded. The MCI patients whose status was not followed for more than 24 months were also removed. The flowchart used to define pMCI and sMCI patients is described in Supplementary Fig. 1. The details and our metabolome tables had limited access to the ADNI project page of the Laboratory of Neuroimage (LONI) (https://ida.loni.usc.edu/login.jsp?project=ADNI).

### Processing of hydrophilic metabolomics data

A total of 1,180 files in mzXML format, containing 905 patients, 245 blanks, and 30 quality controls, were processed using MS-DIAL version 4.7. The MS-DIAL program is designed to provide a metabolome table from untargeted mass spectrometry data with tandem mass spectral libraries such as MassBank and NIST. While many other programs exist, our purpose is to annotate metabolites of the comprehensive spectral libraries containing 39,108 molecules to investigate the relationship between hydrophilic metabolites and AD progressions. The MS-DIAL program was used in this study because of the user-friendly graphical user interface to curate the annotation results. The data processing parameters of minimum amplitude for peak picking and retention time tolerance for peak alignment were set to 100,000 and 0.1 min, respectively, with the minimum amplitude threshold set to detect an average of approximately 3000 peaks per sample in biological samples. Publicly and commercially available mass spectral libraries, containing 1,304,633 spectra of 39,108 compounds, were used for metabolite annotation, which was performed using a spectrum match score cut-off of 0.9 (90%) without retention time information. The annotation results were checked manually.

The hydrophilic metabolome data were exported in a tab-delimited format. The peak heights of all the detected peaks were divided by the peak height of an internal standard, sulfamethoxine (InChIKey = ZZORFUFYDOWNEF-UHFFFAOYSA-N), and multiplied by the average value of the internal standards among the biological samples. Biological samples were excluded if the peak height of the internal standard was not within the average ± 3 × standard deviation (SD) range of the peak heights of the quality control samples. Patients without clinical data were excluded. Unknown peaks were not used in the statistical analyses. Additionally, metabolite peak information was excluded if the peak height values of 30% of the biological samples were less than the mean plus the 3 × SD value of the solvent blank samples. Drugs and their metabolites were excluded from this study. Finally, the profiles of 93 metabolites from 778 patients were used for statistical analysis. Metabolites with the same name and slightly different retention times, indicating the possibility of structural isomers, were distinguished by a series of capital letters, such as metabolite-A and metabolite-B. The lipid nomenclature follows the MS-DIAL lipid nomenclature system or the definition of ADNI dataset (http://prime.psc.riken.jp/compms/msdial/lipidnomenclature.html).

### Statistical analyses

Statistical analysis and data visualization were performed using R language (version 4.1.3). The Mann–Whitney U test with false discovery rate correction was used to calculate *P*-values. To clarify the metabolome differences between pMCI and sMCI, XGBoost was used for machine learning analysis. In addition to the metabolome information, clinical data including age, sex, and APOE4 haplotype labels were included as variables to build a classification model. The auto-parameter tuning function of the package of “tideymodel” package v. 1.0 was used to determine the values of tree depth, min_n, loss_reduction, sample_size, mtry, and learn_rate using tenfold cross validation. The comparison of AUCs and calculation of *P*-values were conducted using the DeLong test. The proportional hazards assumption of each Cox proportional hazard model was confirmed using the Schoenfeld test (Supplementary Table 1). Metabolites that did not meet the proportional hazards assumption were excluded from the analysis of the Cox proportional hazards model. Visualization of the Cox proportional hazard model, calculation of hazard ratios (HR) and *P*-values, and estimation of proportional hazards were performed using the “survival” v.3.2-13 and “survminer” v. 0.4.9 package in R. The optimal cut-off value of the metabolite level in the proportional hazard model assessing the time to progression from MCI to AD was determined at the point closest to the top-left part of the receiver operating characteristic plot representing the highest sensitivity while maintaining specificity. The *P*-value was calculated using the Wald test for each of the proportional hazard models. For the association analysis of single nucleotide polymorphisms (SNPs) and metabolites, we used SNP data that were significantly associated with the progression from MCI to AD in the cohort study by Bellenguez et al. (Supplementary Table 2)^27^, where 71 SNPs data were found in ADNI data. The analysis of metabolites and SNPs and calculation of *P*-values were performed based on a linear regression model adjusted for age and sex using the “lm” function in R.

### Validation of spectral annotation for significantly changed metabolites

Ergothioneine and glycochenodeoxycholic acid were purchased from Cayman Chemical and Santa Cruz Biotechnology, respectively. Sulfamethoxine and oleamide were purchased from the Tokyo Chemical Industry. Liquid chromatography-mass spectrometry (LC–MS)-grade water, acetonitrile, methanol, and formic acid were purchased from Wako.

Liquid chromatography-tandem mass spectrometry (LC–MS/MS) followed the protocol used in the ADNI cohort study^28^, although our equipment was different from the original. The LC system was a Nexera X2 system (Shimadzu, Kyoto, Japan). The standard compounds were separated on Kinetex C18 (100 × 2.1 mm; 1.7 µm; 100 Å) (Phenomenex). The column was maintained at 30 °C at a flow-rate of 0.5 mL/min. The mobile phase consisted of (A) 100% water with 0.1% formic acid and (B) 100% acetonitrile with 0.1% formic acid. Separation was conducted under the following gradient: 0–1 min 5% B, 1–7 min 99.9% B, 7–7.5 min 99.9% B, 7.5–8 min 5% B, and 8–10 min 5%. The temperature of the samples was maintained at 4 °C. The concentration of the standard compounds was adjusted to 1 mM in methanol, and the solvent was transferred to a glass amber vial with a micro insert (Agilent Technologies). A 1 mL aliquot was injected.

Mass spectrometry was performed using a quadrupole time-of-flight mass spectrometer LC–MS-9030 (Shimadzu). The data-dependent acquisition mode was used under the following conditions: interface temperature, 300 °C; ESI spray voltage, 4000 V; MS1 mass range, *m/z* 75–1250; MS2 mass range, *m/z* 50–1250. The various conditions of collision energy, 10, 20, 30, 40, 20 ± 15, 30 ± 15, and 40 ± 15 V were used to obtain a similar spectral pattern to the one in ADNI samples, which have been analyzed using an instrument by ThermoFisher Scientific.

## Result

**Figure 1:**
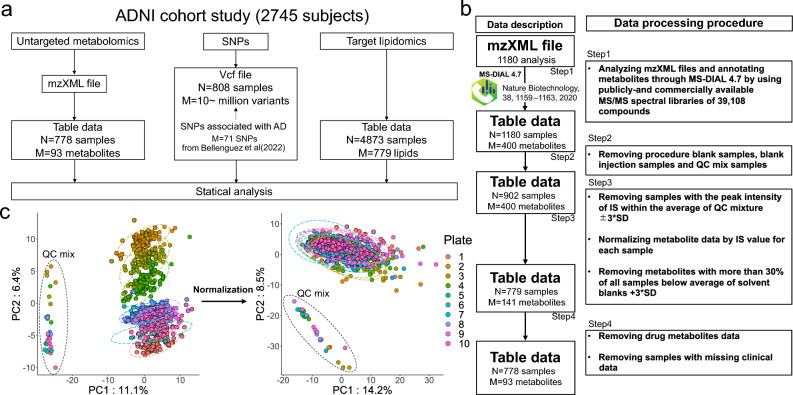
Overview of the multiomics data used in this study. (**a**) A summary of three omics datasets. The N and M values indicate the numbers of participants and variables, respectively, which were used for statistical analyses after several data cleaning steps. (**b**) A summary of data processing methods for the mzXML format files of the untargeted hydrophilic metabolomics. (**c**) The PCA results of an untargeted metabolome table. The left and right panels showing the score plots of the PCA using non-normalized and normalized data, respectively. The term “plate” denotes the number of 96 well plates containing biological samples. The auto-scaling method was applied as the data scaling function.

Three omics datasets including untargeted hydrophilic metabolomics, targeted lipidomics, and SNP data were used for statistical analysis (Fig. 1a). A total of 1180 raw MS data files from untargeted metabolomics were analyzed using MS-DIAL 4, a software program used to generate the metabolome table from MS raw data. We applied several data cleaning steps and obtained the hydrophilic metabolome data containing 93 metabolites from 778 patients (Fig. 1b: see details in the “Materials and methods” section above). A simple data normalization method using the peak height of an internal standard was applied to reduce the batch effects resulting from MS sensitivity drift. Based on the principal component analysis (PCA) score plots, we confirmed that normalization decreased the large variance associated with the batch difference reflected in PC2 of the unnormalized data (Fig. 1c).

### Exploring hydrophilic metabolites associated with AD progression

Hydrophilic metabolomics data from 778 participants were grouped, according to the data labels registered in the ADNI: cognitively normal (CN, n = 163), subjective memory complaints (n = 89), early MCI (n = 259), late MCI (n = 139), and AD (n = 128). Additionally, patients diagnosed with MCI were divided into two groups, pMCI (n = 69) and sMCI (n = 212), to investigate significant metabolites for the prediction of AD progression.

**Figure 2:**
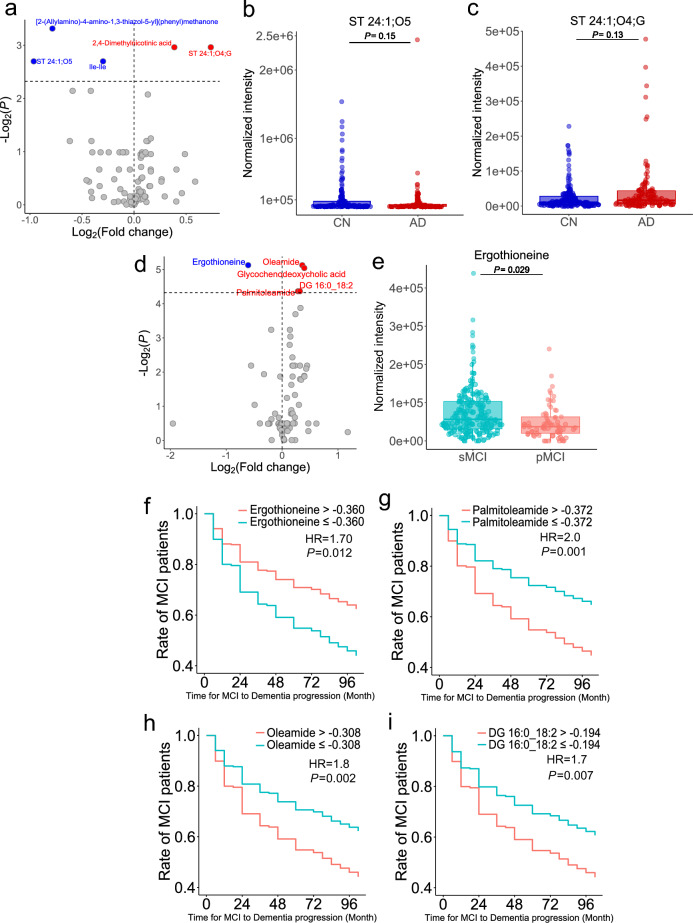
Statistical results of the hydrophilic metabolome data. (**a**) Volcano plot comparing CN and AD. A healthy control was set to the base of the fold change calculation. (**b**,**c**) Box plots of ST 24:1;O5 and ST 24:1;O4;G. (**d**) Volcano plot comparing pMCI and sMCI, with sMCI set as the base for fold change calculation. (**e**) Box plot of ergothioneine. False discovery rate (FDR) correction was used to adjust the *P*-values in (**a**–**e**). (**f**–**i**) Cox proportional hazard model using hydrophilic metabolite information (**f**: ergothioneine, **g**: palmitoleamide, **h**: oleamide, **i**: diacylglycerol (DG) 16:0_18:2). The x- and y-axes show the actual time (month) to diagnosis of dementia from MCI and the ratio (max = 1) showing the remaining MCI patients, respectively. HR, overall hazard ratio.

Five metabolites were highlighted, as judged by *P* < 0.2, as described in the volcano plot showing the fold change and actual *P*-value between CN and AD (Fig. 2a and Supplementary Fig. 2a–c). We observed an increasing trend in ST 24:1;O4;G and a decreasing trend in ST 24:1;O5 levels in AD (Fig. 2b); the ST 24:1;O5 and ST 24:1;O4;G metabolites are cholic acid and a bile acid metabolite of the glycine conjugate. Importantly, changes in conjugated and unconjugated bile acids have also been characterized in the ADNI cohort using the targeted bile acid analysis technique^29^, thereby indicating that our data analysis procedure is applicable to the untargeted metabolomics data of the ADNI. Furthermore, we identified five significantly different metabolites (*P* < 0.05) between the pMCI and sMCI groups (Fig. 2c and Supplementary Fig. 2d–g). Since the retention time information was not used for the original annotations using tandem mass spectral libraries, we confirmed the confidence for ergothioneine, glycochenodeoxycholic acid (GCDCA), and oleamide (Supplementary Fig. 3) by using the authentic standards to validate the retention time values. Ergothioneine, a natural product with antioxidant effects, has been characterized as a significant metabolite in several large cohort studies of dementia and in smaller studies of AD so far (Fig. 2e)^30,31^. According to our investigation, our study is the first to detect ergothioneine with statistical significance in the ADNI cohort and in view of the difference between pMCI and sMCI. While ergothioneine is biosynthesized by basidiomycetes, such as fungi and some bacteria, it is known to be a brain-penetrant antioxidant and cytoprotective agent without pro-oxidant effects. Moreover, ergothioneine can accumulate in human organs, including the brain^32^. Therefore, our findings may provide a rationale for the use of ergothioneine in AD therapy and intervention studies. We further evaluated the four significant metabolites between the pMCI and sMCI groups for use in predicting progression from MCI to the clinical diagnosis of dementia by using the Cox proportional hazard model (Fig. 2f–i, Table 1 and Supplementary Fig. 4). The results showed that patients with lower levels of ergothioneine developed AD earlier, with a 12% higher rate of AD progression within two years (Fig. 2f, HR = 1.70, *P* = 0.012), whereas those with higher levels of palmitoleamide (Fig. 2g, HR = 2.0, *P* = 0.001), oleamide (Fig. 2h, HR = 1.8, *P* = 0.002), and diacylglycerol 16:0_18:2 (Fig. 2i, HR = 1.7, *P* = 0.007) showed the opposite tendency.

**Table 1:** Results of Cox proportional hazard model for hydrophilic metabolome data.

Class	Rate of MCI patients (24 month)	Hazard ratio	*P*-value
Ergothioneine > − 0.360	81%	–	–
Ergothioneine ≤ − 0.360	69%	1.7	0.012
Palmitoleamide ≤ − 0.372	82%	–	–
Palmitoleamide > − 0.372	69%	2	0.001
Oleamide ≤ − 0.308	81%	–	–
Oleamide > − 0.308	69%	1.8	0.002
DG 16:0_18:2 ≤ − 0.194	80%	–	–
DG 16:0_18:2 > − 0.194	69%	1.7	0.007

The rate of MCI patients indicates the rate of those who retained MCI status at 24 months from baseline. A hyphen (–) indicates control. *P*-values and hazard ratios were calculated compared to a control group. *P*-values were calculated using the Wald test.

### Exploring the lipid molecules that associate AD progression

We analyzed 4833 blood lipidome data from 1524 patients divided into several diagnostic groups: CN (n = 1475), MCI (n = 2058), and AD (n = 1300). The MCI group was further divided into pMCI (n = 213) and sMCI (n = 389) groups as defined as above.

**Figure 3:**
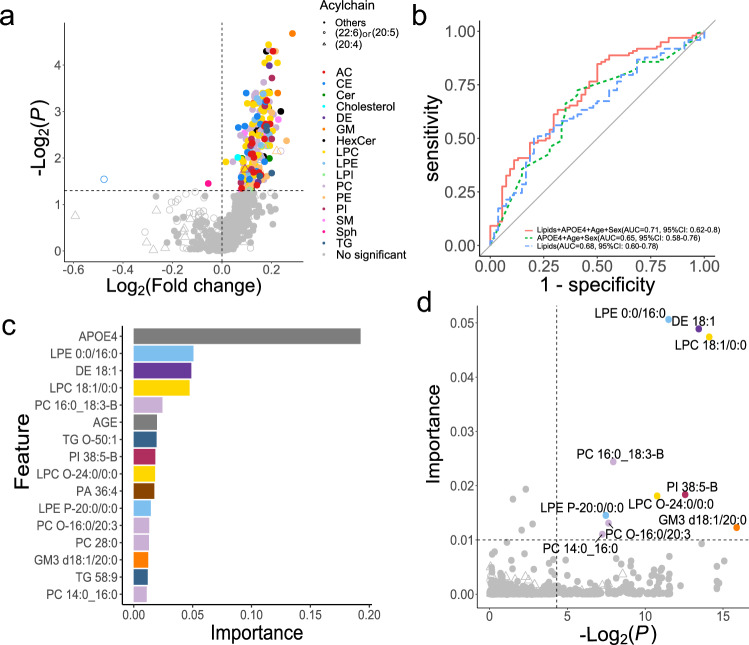
Examination of lipid molecules that changed between pMCI and sMCI. (**a**) The x-axis and y-axis show the log2 fold change and adjusted *P*-value (< 0.05), respectively, with the sMCI value as the denominator. (**b**) Result of the XGBoost machine learning. The receiver operating curves of clinical values, lipid profiles, and both clinical and lipid profiles are described in green, blue, and red, respectively. The area under the curve (AUC) value and 95% confidence interval (95%CI) was also described for each model. (**c**) Important variables in XGBoost using both clinical and lipid profiles. Variables that exceeded 0.01, as variable importance, were described. (**d**) Investigation of the important variables using the results of univariable- and multivariable analyses. The x-axis shows the adjusted *P*-value (< 0.05), which is the result of the Mann–Whitney U test between pMCI and sMCI. The y-axis shows the variable importance of the XGBoost model described in (**c**).

We found an increase in gangliosides (GM1) and ceramides, and a decrease in plasmalogen lipids in patients with AD compared to those in CN patients (Supplementary Fig. 5). Many of these metabolites are associated with the APOE haplotype, which is a major risk factor for AD. This finding is consistent with previous reports^18^. The volcano plot describing the difference between pMCI and sMCI showed that 213 lipid molecules were significantly increased in pMCI (Fig. 3a). Additionally, we used a machine learning method using XGBoost, which aimed to build a model to discriminate pMCI and sMCI patients from baseline lipidome profiles and to extract important metabolite variables for predicting AD progression (Fig. 3b). In addition to lipid profiles, three clinical parameters, including age, sex, and APOE4 number, were used as variables. Importantly, the addition of lipid profiles showed a tendency to improve the performance of the classification model compared with models using only clinical scores, although the difference was not significant (DeLong test:* P* = 0.58), suggesting that lipidome information is a critical factor in achieving a cognitive diagnosis. Based on the metabolites recognized as important variables in the XGBoost model, we found that APOE was the most important variable for predicting AD progression (Fig. 3c). In contrast, lysophosphatidylethanolamine (LPE 0:0/16:0), desmosterol ester (DE 18:1), lysophosphatidylcholine (LPC 18:1/0:0), ganglioside (GM3 d18:1/20:0), and several ether lipids, such as LPC O-24:0/0:0 and LPE P-20:0/0:0 were discovered in this study as important lipid molecules that can be used as markers for discriminant analysis (Fig. 3d). Of these, a previous study using the same ADNI cohort data reported that the expression level of total PE and LPE has the potential to predict AD progression from the MCI stage in which acyl chain properties have not been evaluated^33^.

Modulations of phospholipase A2 (PLA2) activity and expression levels are known phenomena in AD pathology, and the plasmalogen-selective PLA2 is also altered in AD^34–36^. While the lyso-type structure of phospholipids has diverse biological activities as ligands of G protein-coupled receptors (GPCRs), a previous study showed an increase in PLA2 activity in cerebrospinal fluid (CSF) of AD patients, resulting in an increase in LPC^36^. Additionally, the enzymatic activity of phospholipase D (PLD), which metabolizes LPC and LPE to lysophosphatidic acid (LPA), known as the major ligand for six known GPCRs (LPA1–LPA6), is also increased in AD^36,37^. Thus, our results suggest that the significant changes in LPC, LPE, LPC O-, LPE P-, and their diacylglycerol forms reflect the enzymatic dysfunctions in the MCI stage that contribute to the progression to the clinical symptom of AD. Additionally, the activity of the gene expression of the enzyme 24-dehydrocholesterol reductase (DHCR24), which metabolizes desmosterol to cholesterol, is reduced in affected areas of the AD brain^38^. The abnormalities in cholesterol biosynthesis and catabolism are of particular interest for AD therapy. Our results showed that an increase in DE 18:1 would contribute to the hypothesis generation for AD therapy by targeting the enzymes involved in cholesterol metabolism.

**Figure 4:**
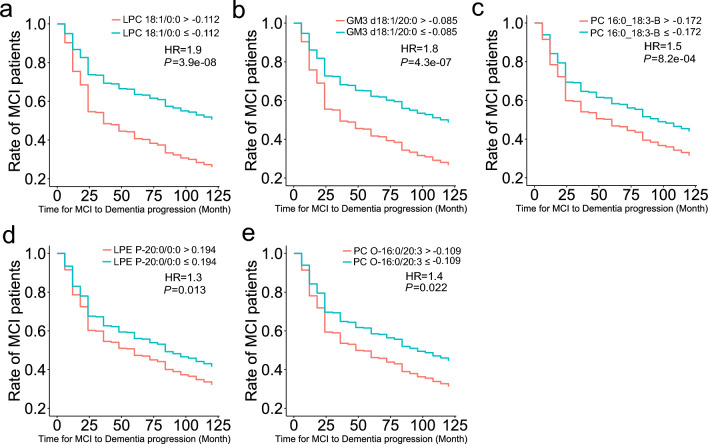
Cox proportional hazard model using the expression cut-off of lipid molecules to predict the time span from MCI to AD. The x- and y-axes show the actual time (month) to diagnosis of dementia from MCI and the ratio (max = 1) of the remaining MCI patients, respectively. HR, overall hazard ratio. (**a**: LPC 18:1/0:0, **b**: GM3 d18:1/20:0, **c**: PC 16:0_18:3-B, **d**: LPE P-20:0/0:0, **e**: PC O-16:0/20:3).

We further evaluated the importance of the significantly altered lipid molecules as biomarkers for predicting the time from MCI to AD onset. The Cox proportional hazard model showed that the expression levels of the five lipid molecules were significant predictors of AD progression (Fig. 4, Table 2, Supplementary Figs. 6 and 7). The most significant metabolite with the highest HR (1.9) was LPC 18:1/0:0, where the higher expression level showed 19% higher rates of AD progression within two years (Fig. 4a, HR = 1.9, *P* = 3.9 × 10^–8^). Higher expression levels of GM3 d18:1/20:0 (Fig. 4b, HR = 1.8, *P* = 4.3 × 10^–7^), PC 16:0_18:3-B (Fig. 4c, HR = 1.50, *P* = 8.2 × 10^–4^), LPE P-20:0/0:0 (Fig. 4d, HR = 1.3, *P* = 0.013), and PC O-16:0/20:3 (Fig. 4e HR = 1.4, P = 0.022) also showed higher rates of AD progression.

**Table 2:** Results of Cox proportional hazard model for lipidome data.

Class	Rate of MCI patients (24 month)	Hazard ratio	*P*-value
LPC 18:1/0:0 ≤ − 0.112	74%	–	–
LPC 18:1/0:0 > − 0.112	55%	1.9	3.9e−8
GM3 d18:1/20:0 ≤ − 0.085	73%	–	–
GM3 d18:1/20:0 > − 0.085	56%	1.8	4.3e−7
PC 16:0_18:3-B ≤ − 0.172	69%	–	–
PC 16:0_18:3-B > − 0.172	60%	1.5	8.2e−04
LPE P-20:0/0:0 ≤ 0.194	68%	–	–
LPE P-20:0/0:0 > 0.194	59%	1.3	0.013
PC O-16:0/20:3 ≤ − 0.109	70%	–	–
PC O-16:0/20:3 > − 0.109	59%	1.4	0.022

The rate of MCI patients indicates the rate of those who retained MCI status at 24 months from baseline. A hyphen (–) indicates control. *P*-values and hazard ratios were calculated compared to a control group. *P*-values were calculated using the Wald test.

### Using SNPs data to explore the association between the genome and metabolome information

**Figure 5:**
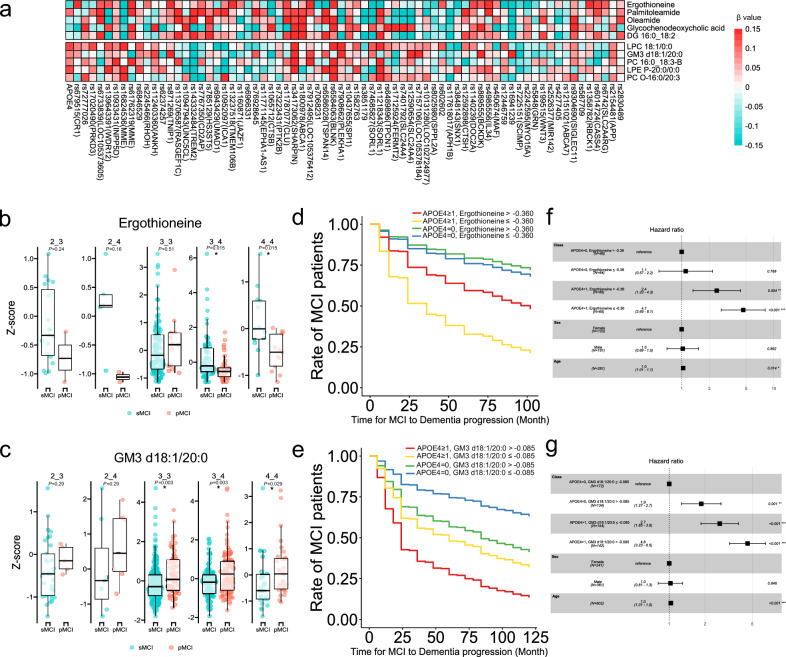
Summary of SNP data and their correlation with the metabolome. (**a**) Heatmap analysis showing the association between metabolites and SNPs related to AD progression. (**b**,**c**) Box plots of ergothioneine and GM3 d18:1/20:0, respectively, with *P*-values adjusted for the false discovery rate in each APOE haplotype. (**d**,**e**) The Cox proportional hazards model predicting the time from MCI to AD by integrating the expression cut-off values of hydrophilic metabolites and lipid molecules and the number of APOE4 alleles present (**d**: ergothioneine, **e**: GM3 d18:1/20:0). We conducted comparisons within patient groups defined by APOE4 status and metabolite levels. The x-axis represents the actual time from MCI to the diagnosis of dementia (months), and the y-axis represents the remaining proportion of MCI patients (max = 1). (**f**,**g**) Hazard ratios of the Cox proportional models. *P*-values and hazard ratios were calculated by comparing each group of patients with a reference group (reference groups exhibited no APOE4 alleles and had high ergothioneine levels (**f**) and no APOE4 alleles and low GM3 d18:1/20:0 levels (**g**)). Integration of APOE4 allele status and metabolite information resulted in an improved predictive ability for AD progression.

We investigated the association between metabolites levels at the MCI stage and risk alleles for which associations with AD progression from MCI have been discovered in previous genome-wide association studies (GWAS)^27^, because the statistical power in ADNI cohort is lower than that of the other studies due to the limited number of patients examined. We investigated the correlation between metabolites and 71 SNPs exhibiting risk for AD progression in a linear regression model (Supplementary Table 2). The results of the metabolite GWAS showed few associations between metabolites and genotypes (Fig. 5a).

Moreover, we compared metabolites levels between pMCI and sMCI for each APOE haplotype (Fig. 5b and c, Supplementary Fig. 8). The results showed a decrease in ergothioneine and an increase in GM3 18:1/20:0 in pMCI regardless of the APOE haplotype (Fig. 5b and c). Additionally, the Cox proportional hazards model only using the number of APOE4 alleles in the ADNI cohort showed that AD progression levels in MCI patients can be stratified (Supplementary Fig. 9). The Cox proportional hazards model using metabolite levels and the number of APOE4 alleles showed that patients with MCI with low blood levels of ergothioneine and even one APOE4 allele had a 35% higher rate of AD progression within two years than those with low blood levels of ergothioneine and no APOE4 allele (HR = 4.7, *P* = 3.7 × 10^–8^). Moreover, patients with MCI, high GM3 d18:1/20:0 blood levels, and at least one APOE4 allele also had a 40% higher rate of AD progression within two years than those with low blood levels of GM3 d18:1/20:0 and no APOE4 allele (HR = 4.6, P = 2.3 × 10^–16^) (Fig. 5d–g and Table 3). These data demonstrate that by combining APOE4 allele data with metabolites data can help to identify patients with a high rate of AD progression (Supplementary Fig. 10). These results suggest that metabolite information can complement genomic data for predicting the onset of AD.

**Table 3:** Results of Cox proportional model for metabolites and number of APOE4 alleles.

Class	Rate of MCI patients (24 month)	Hazard ratio	*P*-value
APOE4 = 0, ergothioneine > − 0.360	87%	–	–
APOE4 = 0, ergothioneine ≤ − 0.360	85%	1.1	0.77
APOE4 ≥ 1, ergothioneine > − 0.360	73%	2.4	0.0038
APOE4 ≥ 1, ergothioneine ≤ − 0.360	52%	4.7	3.7e−08
APOE4 = 0, GM3 d18:1/20:0 ≤ − 0.085	83%	–	–
APOE4 = 0, GM3 d18:1/20:0 > − 0.085	69%	1.9	0.001
APOE4 ≥ 1, GM3 d18:1/20:0 ≤ − 0.085	62%	2.7	7.5e-07
APOE4 ≥ 1, GM3 d18:1/20:0 > − 0.085	43%	4.6	2.3e-16

The rate of MCI patients indicates the rate of those who retained MCI status at 24 months from baseline. A hyphen (–) indicates control. *P*-values and hazard ratios were calculated compared to a control group. *P*-values were calculated using the Wald test.

## Discussion

We investigated the importance of metabolomic information in the prediction of AD onset using the ADNI data. The MS data for hydrophilic metabolome profiling were reanalyzed using the MS-DIAL program, followed by data curation to exclude MS sensitivity drift and drug-related metabolite information. To the best of our knowledge, this is the first study to report the detail of the hydrophilic metabolomes of 778 patients registered in the ADNI repository.

Five hydrophilic metabolites at baseline were significantly different between sMCI and pMCI. The significance of the metabolites was evaluated by survival analysis. Among these, the expression level of ergothioneine, a natural amino thione with potent antioxidant and cytoprotective activities^32^, was significantly lower in patients with pMCI. Ergothioneine accumulates in animals and plants, and can be biosynthesized in actinomycetota, such as *Mycobacterium smegmatis*, and a proportion of fungi, such as *Neurospora crassa*. In the human body, large amounts of ergothioneine are found in the erythrocytes, eyes, semen, and skin^32^. Our study is the first to show the statistically significant difference in ergothioneine between pMCI and sMCI, and by the survival analysis in the ADNI cohort. Other cohort studies have reported that blood ergothioneine levels decrease with cognitive decline and dementia^30,31^. Importantly, our study highlights the importance of considering blood ergothioneine levels to stratify the clinical stages of sMCI and pMCI and predict the rate of AD progression.

Increased oleamide and GCDCA were also observed in patients with pMCI. Oleamide is classified as a primary amide of fatty acids, and is known to act as a ligand for cannabinoid receptors type 1 and 2 (CB1 and CB2), which regulate the brain and central nervous system^39^. Activation of the endocannabinoid system via CB1 and CB2 has neuroprotective effects by inhibiting the release of presynaptic neurotransmitters^40^. Thus, the increase of oleamide in pMCI suggests activation of oleamide biosynthesis and may reflect metabolic adaptation to increased neurotoxicity in AD pathology.

The lipidomic results showed that many lipids were elevated in patients with pMCI, and 10 biomolecules were selected by the machine learning method as biomarker candidates for predicting AD progression. Significant increases in the levels of lysophospholipids, including LPE 0:0/16:0, LPC 18:1/0:0, LPC O-24:0/0:0, and LPE P-20:0/0:0, were observed. Previous studies using the same ADNI cohort data have also reported that total PE and LPE levels can be used to predict AD progression from the MCI stage^33^. The dysregulation of PLA2 expression and bioactivity is observed in AD pathology^35,36^, and plasmalogen specific PLA2 is also altered in AD^37^. The lysophospholipids have various biological activities as ligands for GPCRs, and a previous study has shown that PLA2 activity is increased in the CSF of AD patients, resulting in the increase of LPC molecules^36^. The enzymatic activity of PLD, which metabolizes LPC and LPE to LPA, is also increased in AD^36^. While the association between the acyl chain property of LPA and GPCRs (LPA1–6) has been studied, the LPA lipid containing oleic acid (LPA 18:1/0:0) at the *sn1* position is known to be the ligand for LPA4 expressed in the brain^37^, but its biological role in AD remains unknown. Understanding the regulatory mechanisms of phospholipids and their acyl chain properties is an emerging need, as each lipid molecule has distinct biological importance in maintaining brain homeostasis.

Additionally, DE 18:1 and GM3 d18:1/20:0 were important signatures of pMCI. The enzymatic activity of DHCR24, which converts desmosterol to cholesterol, is impaired in the brain of AD patients^38^. As the dysfunction of cholesterol biosynthesis and catabolism is of particular interest for the treatment of AD, our result, which shows an increase in DE 18:1, may drive the generation of hypotheses for AD therapy. The GM3 gangliosides are sphingoglycolipids that are abundant in the central nervus system. Gangliosides are elevated in the brain in AD, and are thought to be involved in Aβ aggregation and amyloid plaque formation^41^. Therefore, the elevated plasma GM3 levels in pMCI may reflect the elevated brain levels of gangliosides in AD pathology.

Furthermore, metabolites, such as ergothioneine and GM3 d18:1/20:0, varied significantly in pMCI, regardless of the APOE haplotype, and combining metabolite information with APOE4 possession improved the stratification performance of MCI patients with faster AD progression, thereby suggesting that these metabolites would be useful signatures for stratifying patients who cannot be distinguished using the APOE loci. This study characterized several important metabolites predicting AD progression from MCI. Although the cohort in this study was relatively large when compared to other AD studies dealing with metabolomics data^30^, the sample size is very small when compared to GWAS studies^27^ involving genome data, thus decreasing the statistical power. Therefore, the results should be validated in an independent cohort.

## Conclusions

We performed a multiomics analysis using untargeted hydrophilic metabolomic, lipidomic, and SNP data to investigate the relationship between metabolites, genotypes, and phenotypes. Our data analysis procedure characterized several important metabolites that may predict the progression of AD from MCI, which will contribute to the development of biomarkers for the early detection of brain pathology. Furthermore, these findings would contribute, at least in part, to our understanding of the mechanisms of MCI in AD progression. Most importantly, this study demonstrates that the re-analysis of large-scale MS data can provide new insights into diseases for which there is still no effective treatment or diagnosis.

### Supplementary Information

Supplementary Figures.

Supplementary Tables.

## Data Availability

The mass spectrometry raw data and the metabolome table created in this study had limited access to the ADNI project page of the Laboratory of Neuroimage (LONI) (https://ida.loni.usc.edu/login.jsp?project=ADNI).
