# Supplementary material for: Multiomics analysis to explore blood metabolite biomarkers in an Alzheimer’s Disease Neuroimaging Initiative cohort
Source: Sci Rep. 2024 Apr 2;14:6797. doi: 10.1038/s41598-024-56837-1 (PMC10987653; doi:10.1038/s41598-024-56837-1)
Supplement: Supplementary file 1 — Supplementary Figures. [file 41598_2024_56837_MOESM1_ESM.docx]

**Supplementary Information**

**Multiomics analysis to explore blood metabolite biomarkers in an Alzheimer’s Disease Neuroimaging Initiative cohort**

Takaki Oka^1^, Yuki Matsuzawa^1^, Momoka Tsuneyoshi^2^, Yoshitaka Nakamura^2^, Ken Aoshima^3,4^, Hiroshi Tsugawa^1,5,6,7^ for the Alzheimer’s Disease Metabolomics Consortium**

1. Department of Biotechnology and Life Science, Tokyo University of Agriculture and Technology, Tokyo, Japan
2. Human Biology Integration Foundation, Eisai Co., Ltd., Ibaraki, Japan
3. Microbes & Host Defense Domain, Eisai Co., Ltd., Ibaraki, Japan
4. School of Integrative and Global Majors, University of Tsukuba, Ibaraki, Japan
5. RIKEN Center for Sustainable Resource Science, Yokohama, Japan
6. RIKEN Center for Integrative Medical Sciences, Yokohama, Japan
7. Graduate School of Medical Life Science, Yokohama City University, Yokohama, Japan

**Data used in preparation of this article were generated by the Alzheimer’s Disease Metabolomics Consortium

**Corresponding authors**

Hiroshi Tsugawa: htsugawa@go.tuat.ac.jp

**Contents:**

Supplementary Figures 1-10

**Supplementary Figures**


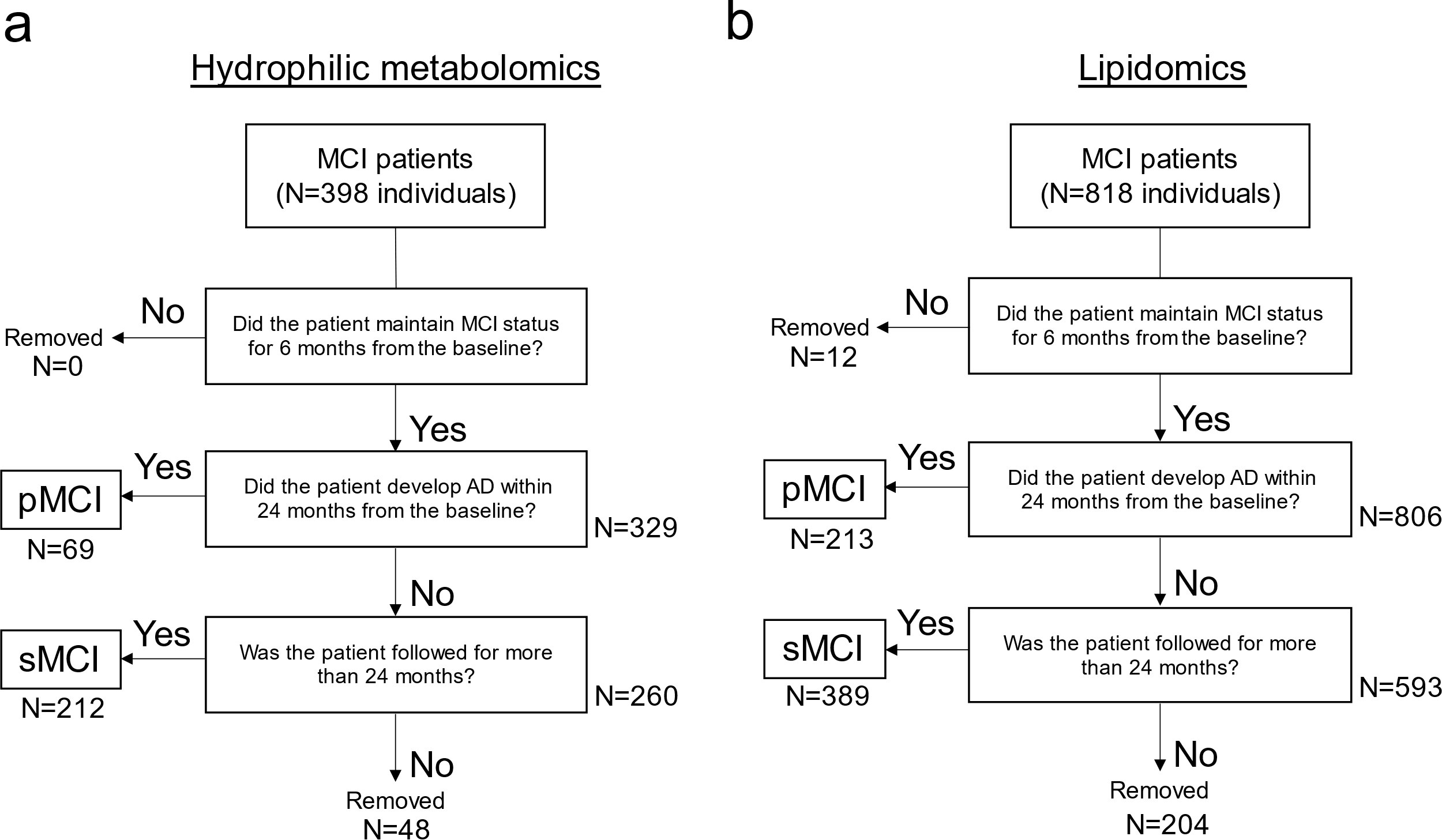


**Supplementary Figure 1. Flowchart of classification of MCI patients.** Patients diagnosed with MCI at baseline who developed AD from six months by two years were defined as having progressive MCI (pMCI), and the others were classified as having sustained MCI (sMCI). Data from patients diagnosed with AD within six months from the baseline were excluded. The MCI patients whose status was not followed for more than 24 months were also removed. The N values indicate the numbers of patients (**a**: hydrophilic metabolomics data, **b**: lipidomics data).


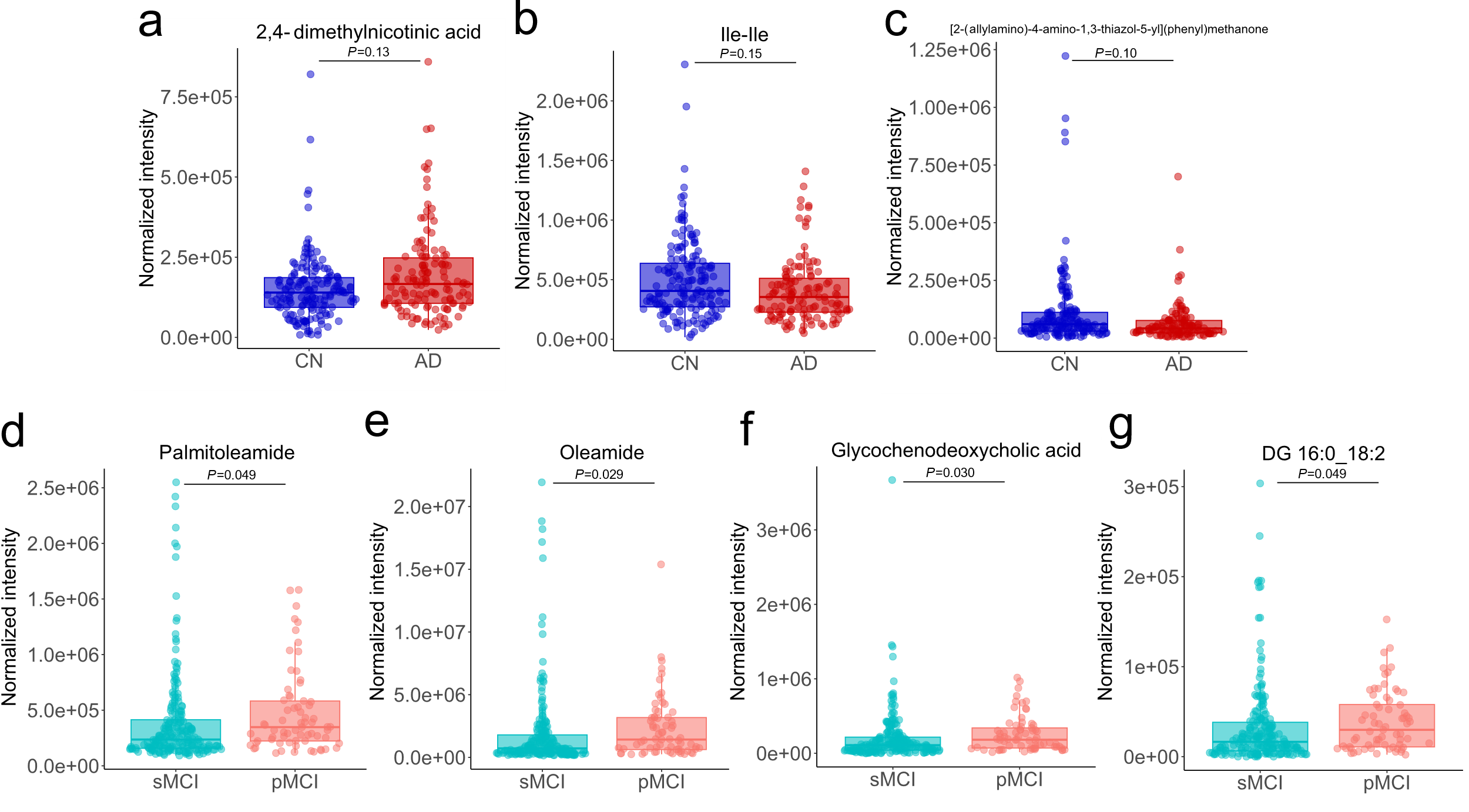


**Supplementary Figure 2. Comparison of significant hydrophilic metabolites.** (**a**-**c**) Boxplot of metabolites exhibiting variable trends between AD and CN (**a**: 2,4-dimetilnicotinic acid, **b**: isoleucine-isoleucine, **c**: [2-(Allylamino)-4-amino-1,3-thiazol-5-yl](phenyl)methanone). (**d**-**g**) Boxplot of metabolites showing a significant difference between pMCI and sMCI (**d**: palmitoleamide, **e**: oleamide, **f**: glycochenodeoxycholic acid, **g**: DG 16:0_18:2). *P*-values were calculated using the Mann-Whitney U test. False discovery rate correction was used to adjust the *P*-values.

**
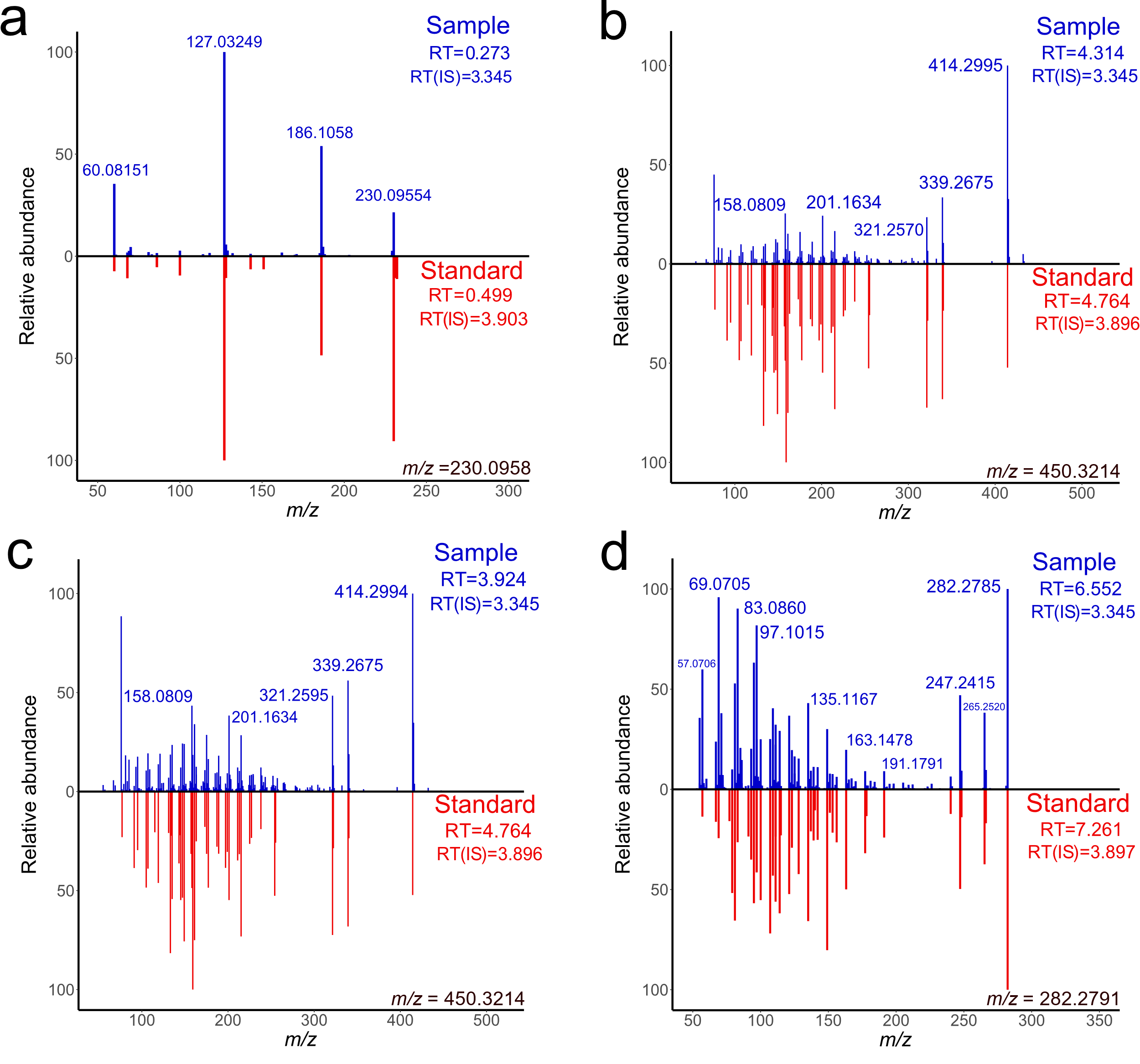
**

**Supplementary Figure 3. Comparison of MS/MS spectra from ADNI samples and authentic standards.** The ADNI’s experimental and standard spectra for (a) ergothioneine, (b) glycochenodeoxycholic acid-A, (c) glycochenodeoxycholic acid-B, and (d) oleamide were described in the upper (blue) and lower (red) panels. Retention time (RT) of analytes and the RT value of sulfamethoxine; RT (IS) used as the internal standard in the ADNI study are described. The precursor *m/z* of standards is shown at the right-bottom area of each figure.


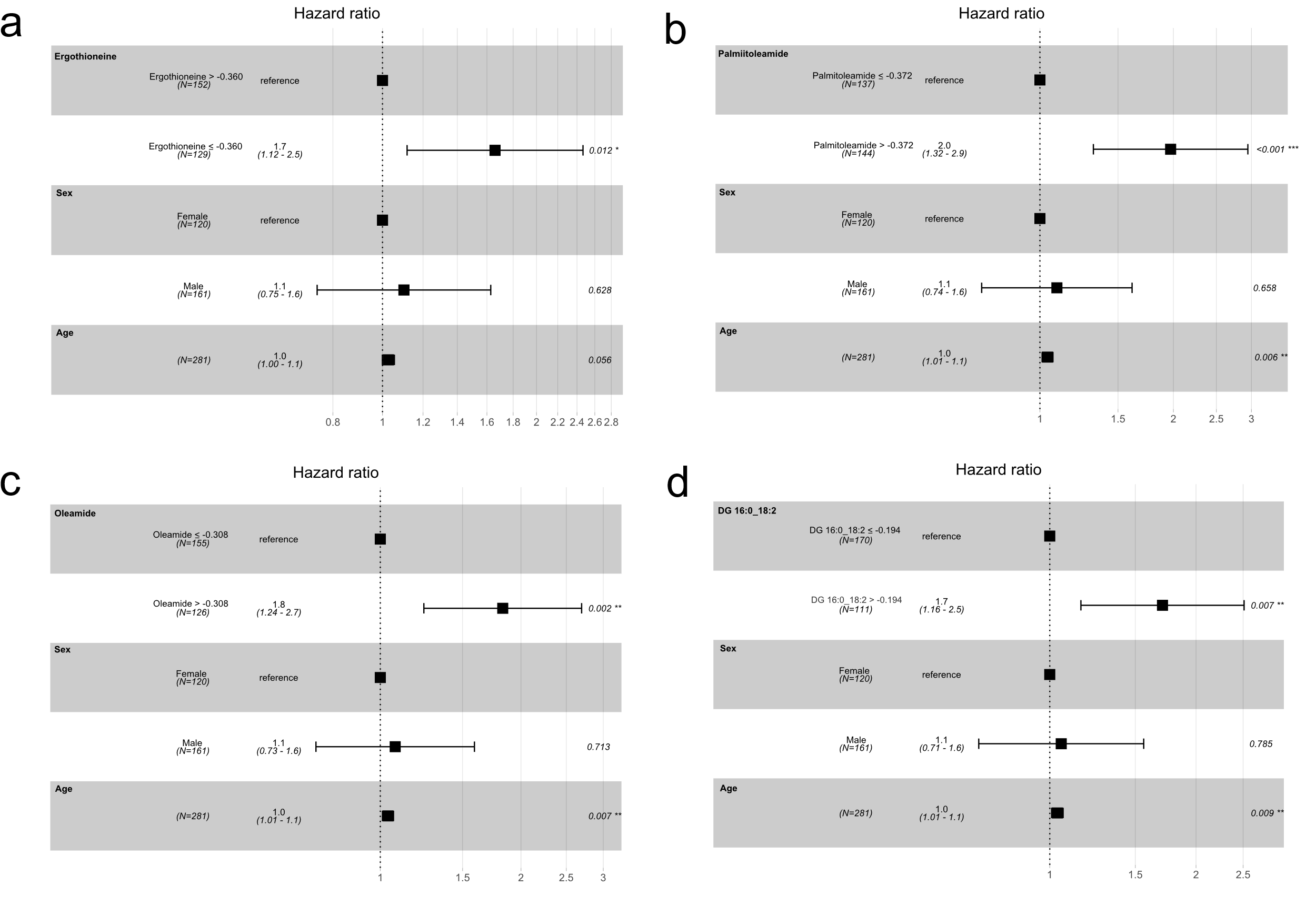


**Supplementary Figure 4.** **Hazard ratios of Cox proportional hazards models for hydrophilic metabolite levels** (**a**: ergothioneine, **b**: palmitoleamide, **c**: oleamide, **d**: DG 16:0_18:2). Cox proportional hazards models were adjusted for age and sex as covariates. Hazard ratios and *P*-values were calculated for each group with respect to the reference group. *P*-values were calculated using the Wald test.


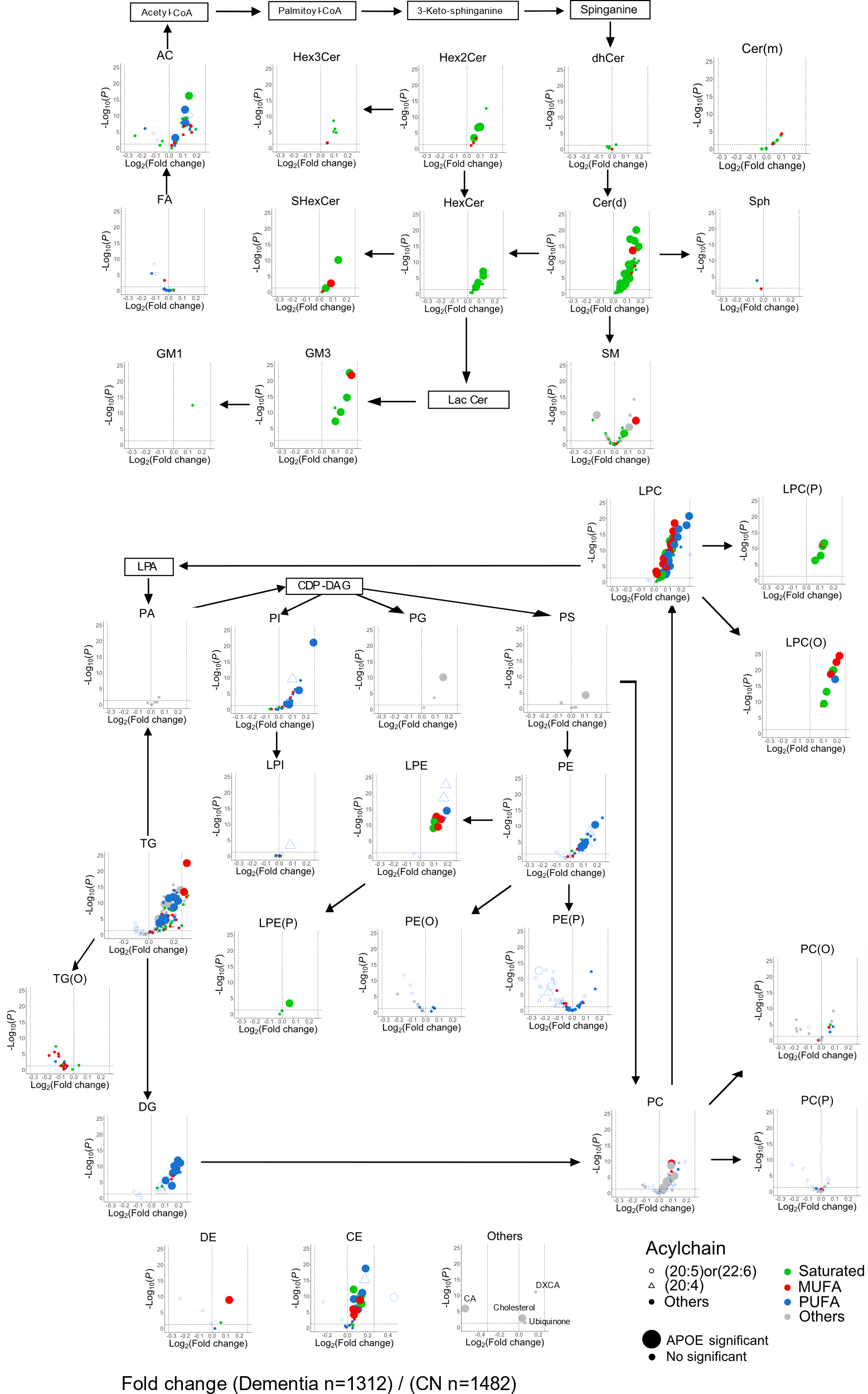
**Supplementary Figure 5. Comparison of lipids between AD and CN.** The healthy control (CN) was set to the base of the fold change calculation. *P*-values were calculated using the Mann-Whitney U test. False discovery rate correction was used to adjust the *P*-values. The x- and y-axes show the log_2_ fold change- and -log_10_ *P*-value. The horizontal dot line indicates the value of *P*=0.05. The fatty acid composition in complex lipids was described by the different plot shape. The lipids containing docosahexaenoic acid (22:6) or eicosapentaenoic acid (20:5), arachidonic acid (20:4), and others were described by no-filled circle, triangle, and filled circle, respectively. The lipid containing saturated, monounsaturated (MUFA), and polyunsaturated fatty acids (PUFA) are distinguished by plot colors of green, red, and blue, respectively. Significantly changed metabolites in APOE4/4 patients when compared to the genotypes are indicated by the larger circle symbol.


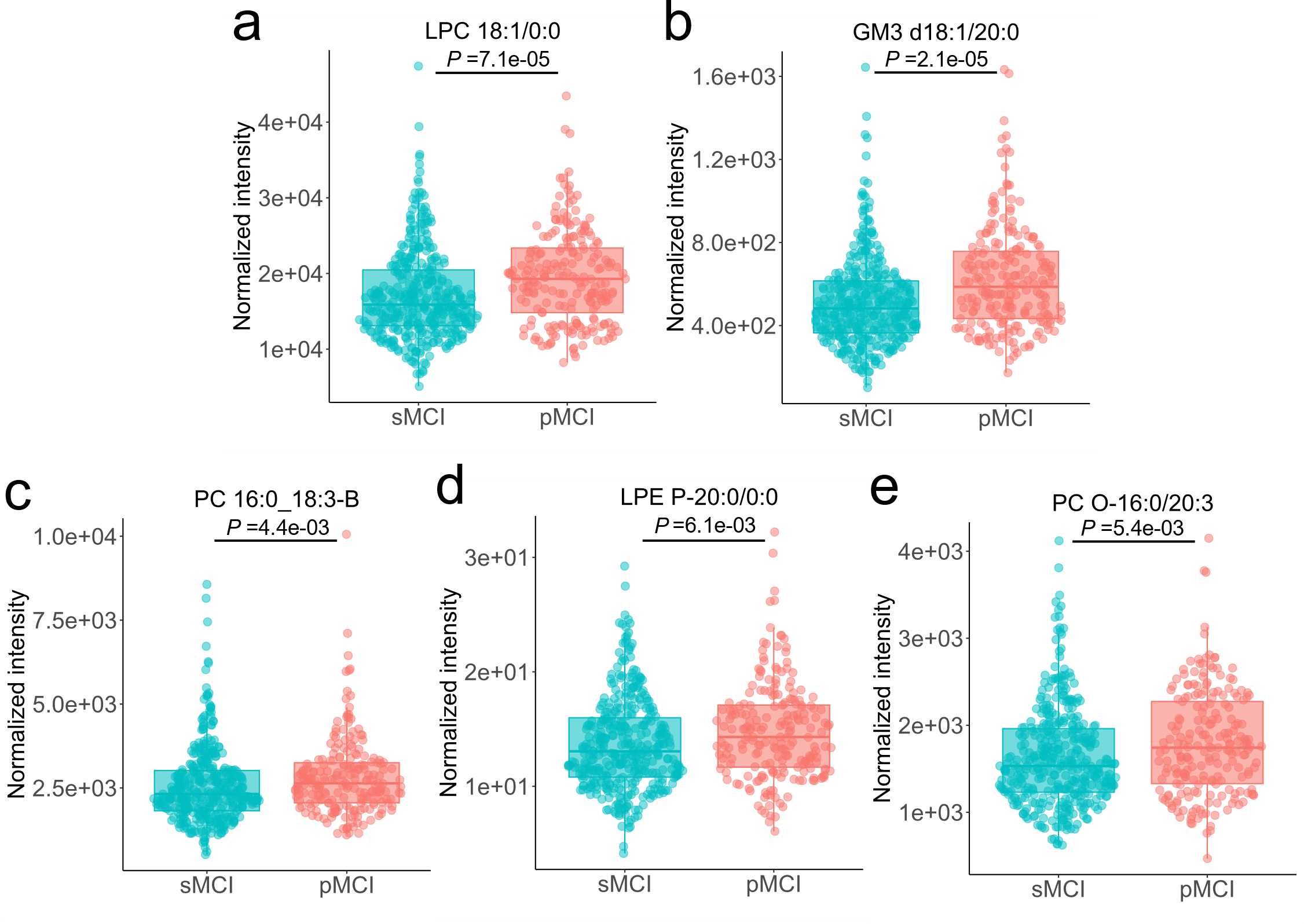


**Supplementary Figure 6. Comparison of significant lipid molecules.** (**a**-**e**) Boxplot of metabolites exhibiting variable trends between pMCI and sMCI (**a**: LPC 18:1/0:0, **b**: GM3 d18:1/20:0, **c**: PC 16:0_18:3-B, **d**: LPE P-20:0/0:0, **e**: PC O-16:0/20:3). *P*-values were calculated using the Mann-Whitney U test. False discovery rate correction was used to adjust the *P*-values.


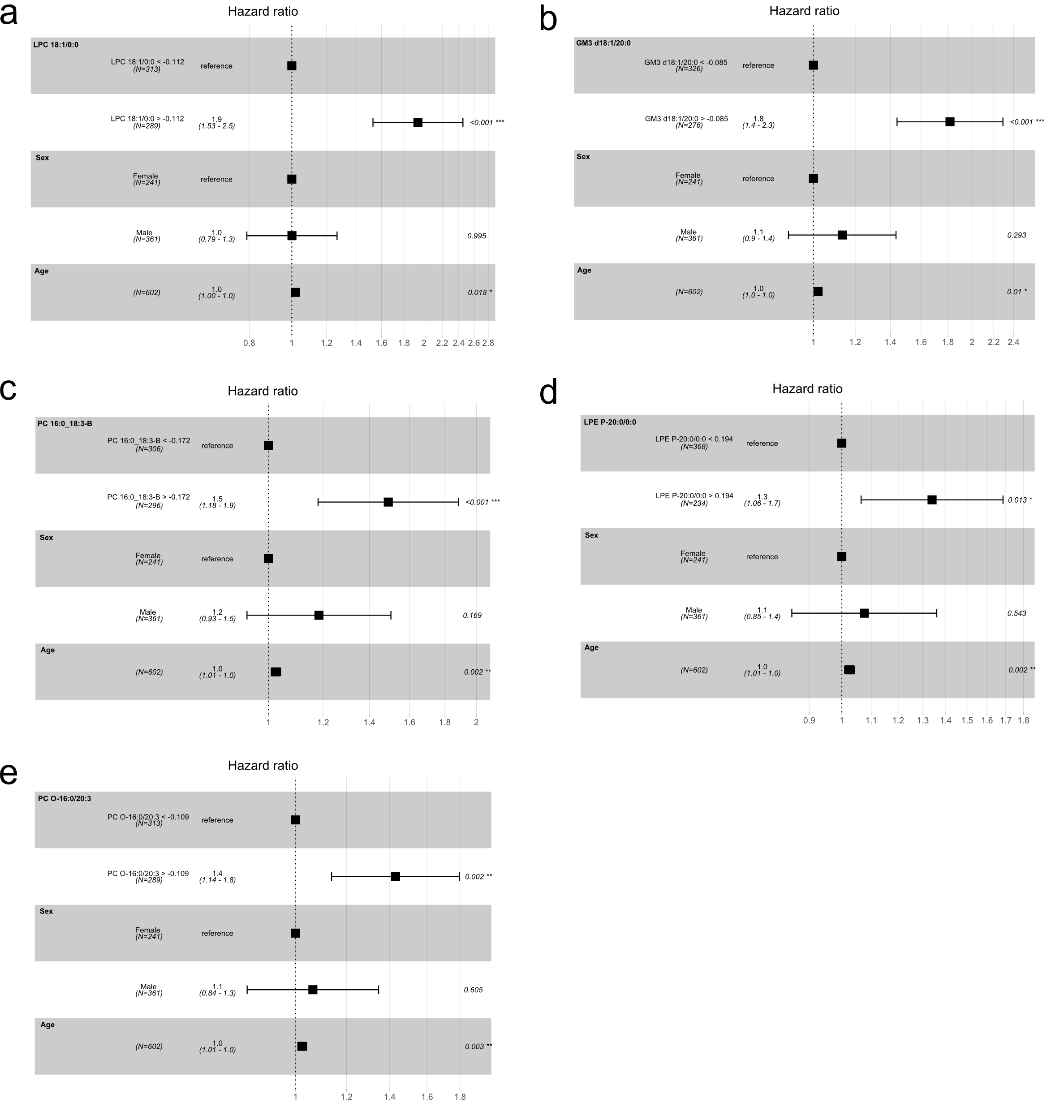


**Supplementary Figure 7.** **Hazard ratios of Cox proportional hazards model for lipid molecule levels** (**a**: LPC 18:1/0:0, **b**: GM3 d18:1/20:0, **c**: PC 16:0_18:3-B, **d**: LPE P-20:0/0:0, **e**: PC O-16:0/20:3). Cox proportional hazards models were adjusted for age and sex as covariates. Hazard ratios and *P*-values were calculated for each group with respect to the reference group. *P*-values were calculated using the Wald test.


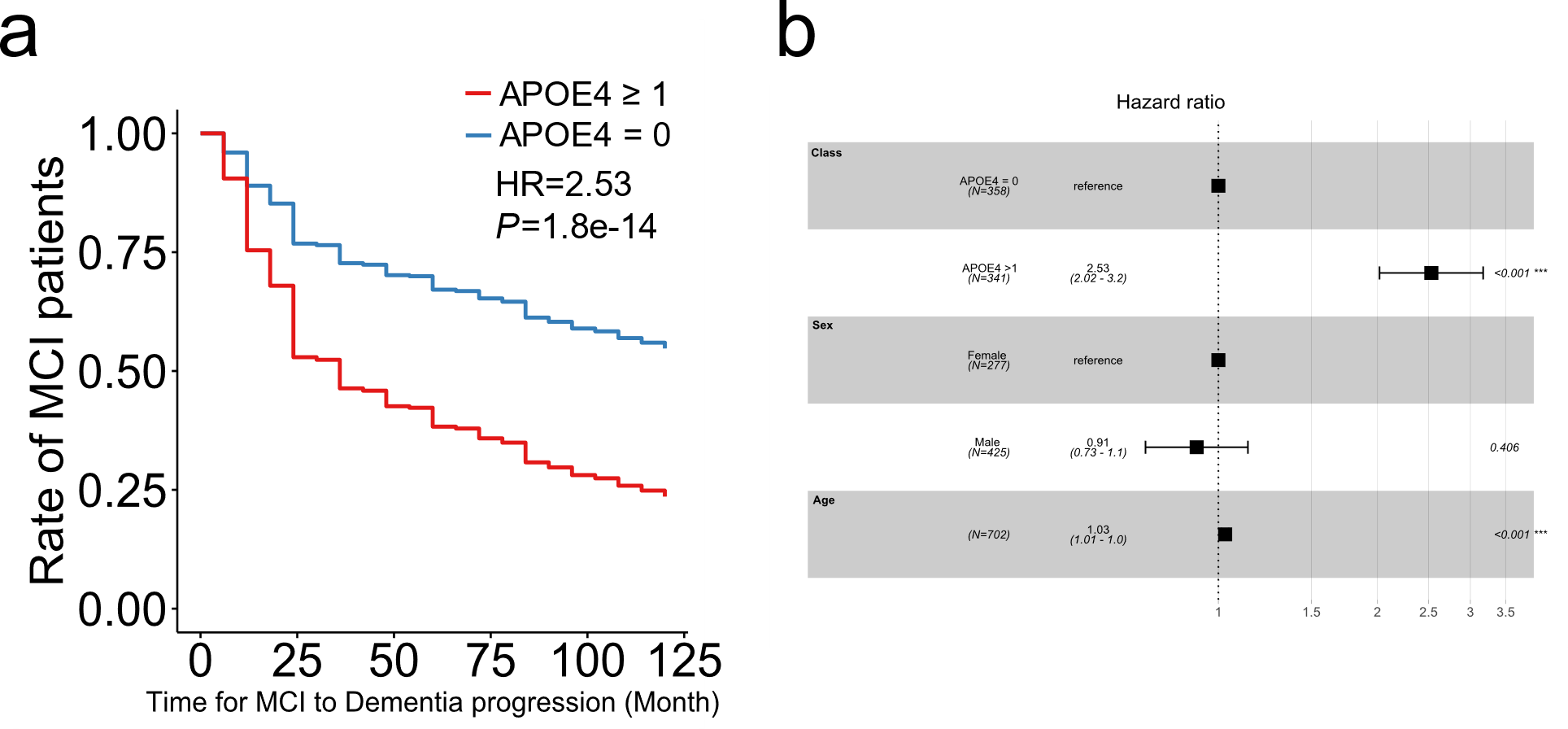


**Supplementary Figure 8. Summary of stratification of MCI patients to predict the time span from MCI to AD.** (**a**) Cox proportional hazard models using the number of APOE4 alleles present to predict the time span from MCI to AD. The x-axis represents the actual time from MCI to the diagnosis of dementia (months), and the y-axis represents the remaining proportion of MCI patients (max=1). (**b**) Hazard ratios of the Cox proportional hazard models using the number of APOE4 alleles. Hazard ratios and *P*-values were calculated for the APOE4 ≥ 1 group compared to the APOE4 = 0 group. *P*-values were calculated using the Wald test.

**
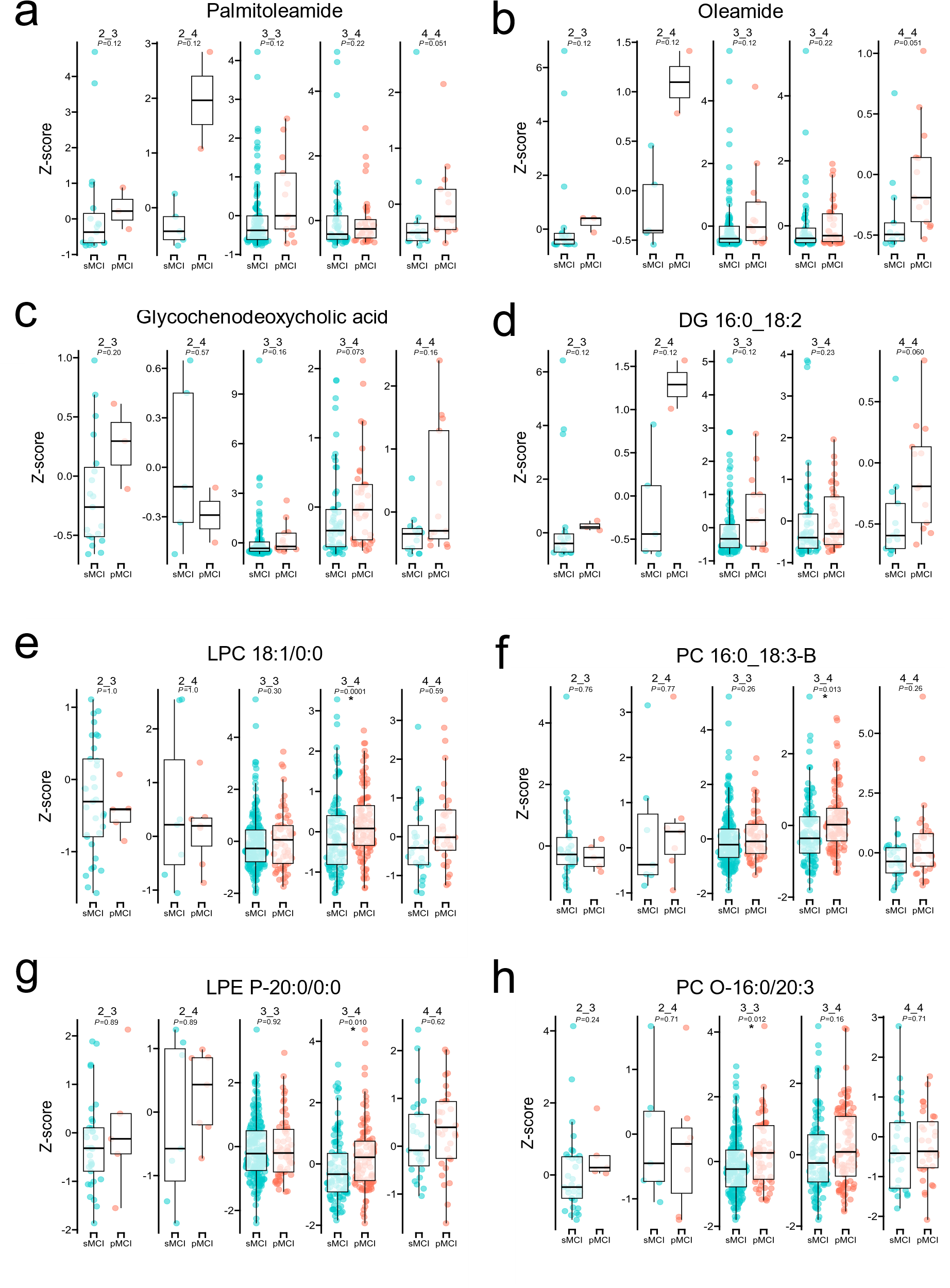
**

**Supplementary Figure 9. Box plots of hydrophilic metabolites and lipid molecules in the pMCI and sMCI groups for each APOE haplotype.** (**a**-**d**) Boxplot of hydrophilic metabolites (**a**: palmitoleamide, **b**: oleamide, **c**: glycochenodeoxycholic acid, **d**: DG 16:0_18:2). (**e**-**h**) Boxplot of lipid molecules (**e**: LPC 18:1/0:0, **f**: PC 16:0/18:3-B, **g**: LPE P-20:0/0:0, **h**: PC O-16:0/20:3). *P*-values were calculated using the Mann-Whitney U test. False discovery rate correction was used to adjust the *P*-values. * denotes adjusted *P*-values < 0.05. The results showed trends of metabolite variations in pMCI regardless of the APOE haplotype.


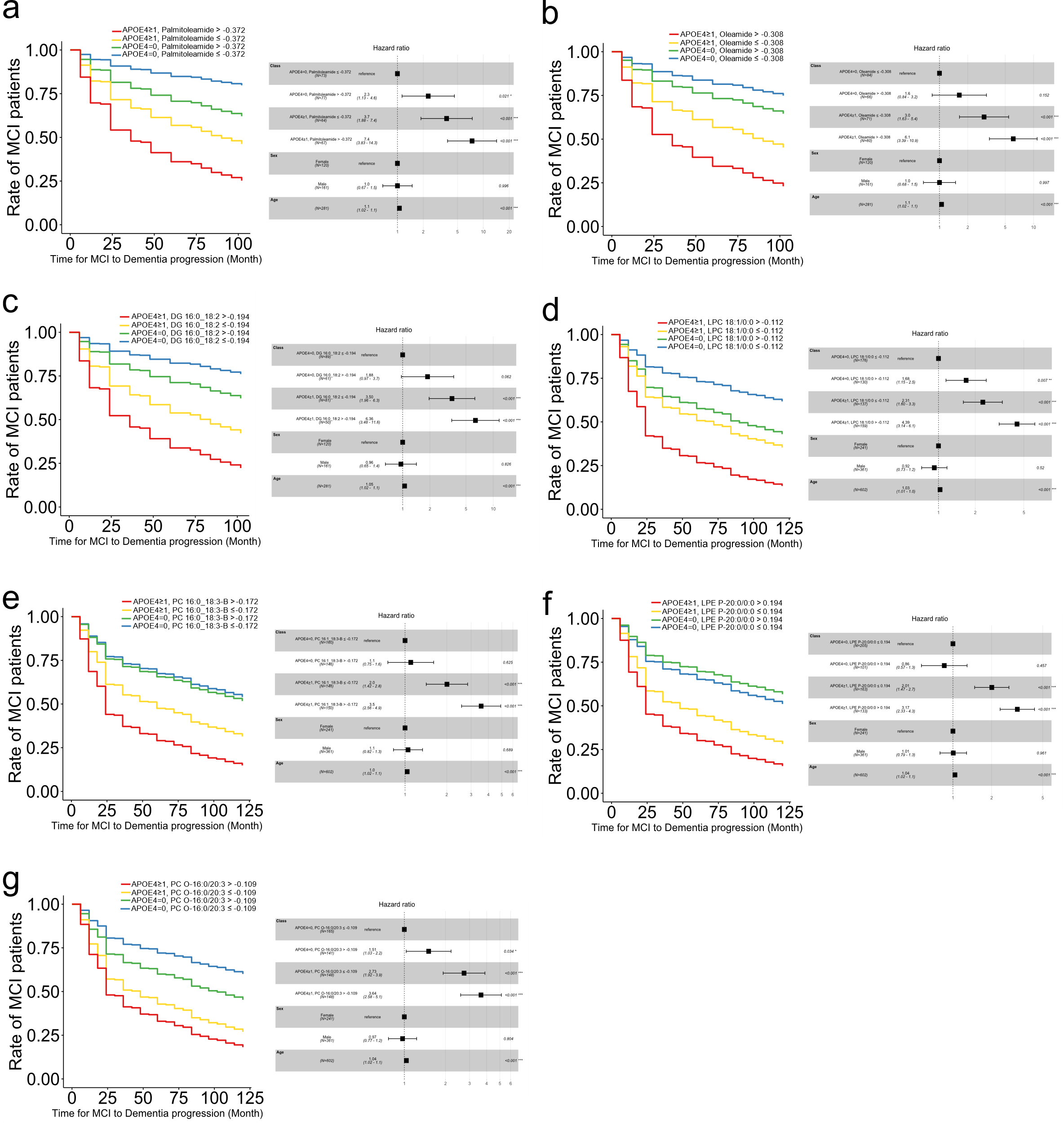


**Supplementary Figure 10. Cox proportional hazards models predicting the time from MCI to AD by integrating the expression cut-off values of hydrophilic metabolites and lipid molecules and the number of APOE4 alleles.** The x-axis represents the actual time from MCI to the diagnosis of dementia (months), and the y-axis represents the remaining proportion of MCI patients (max=1). HR, overall hazard ratio. *P*-values and hazard ratios were calculated compared to a reference group. *P*-values were calculated using the Wald test (**a**: palmitoleamide, **b**: oleamide, **c**: DG 16:0_18:2, **d**: LPC 18:0/0:0, **e**: PC 16:0_18:3-B, **f**: LPE P-20:0/0:0, **g**: PC O-16:0_20:3).
